# Engineering of Hybrid Nanoporous Anodic Alumina Photonic Crystals by Heterogeneous Pulse Anodization

**DOI:** 10.1038/s41598-018-27775-6

**Published:** 2018-06-21

**Authors:** Siew Yee Lim, Cheryl Suwen Law, Lluís F. Marsal, Abel Santos

**Affiliations:** 1 School of Chemical Engineering, The University of Adelaide, 5005 Adelaide, Australia; 2 Institute for Photonics and Advanced Sensing (IPAS), The University of Adelaide, 5005 Adelaide, Australia; 3 ARC Centre of Excellence for Nanoscale BioPhotonics (CNBP), The University of Adelaide, 5005 Adelaide, Australia; 4 Department of Electronic, Electric, and Automatics Engineering, Universitat Rovira i Virgili, 43007 Tarragona, Spain

## Abstract

In this study, we present an advanced nanofabrication approach, so-called ‘heterogeneous pulse anodization’ (HPA), in which galvanostatic stepwise and apodized sinusoidal pulse anodizations are combined in a single process. This novel anodization method enables the precise optical engineering of the characteristic photonic stopbands (PSBs) of nanoporous anodic alumina photonic crystals (NAA-PCs). The resulting structures are hybrid PCs (Hy-NAA-PCs) composed of distributed Bragg reflectors (DBRs) and apodized gradient-index filters (APO-GIFs) embedded within the same PC structure. The modification of various anodization parameters such as anodization period, relative and total anodization time, structural arrangement of PCs within Hy-NAA-PCs, and pore widening time allows the fine-tuning of the PSBs’ features (i.e. number, position and bandwidth of central wavelength) across the spectral regions. The effects of these fabrication parameters are systematically assessed, revealing that the positions of the characteristic transmission bands of Hy-NAA-PCs are highly controllable. Our study provides a comprehensive rationale towards the development of unique Hy-NAA-PCs with controllable optical properties, which could open new opportunities for a plethora of applications.

## Introduction

Photonic crystals (PCs) are periodic optical nanostructures that can control the propagation of light by modifying the motion of incoming photons when these travel across the PC’s structure^1–5^. These structures feature a dimensional variation in refractive index, which can be arranged a 1D, 2D or 3D fashion^6^. PCs are extensively used in a broad range of applications, including telecommunications^7,8^, quantum computing^9,10^, energy and water remediation^11–17^, medical imaging^18,19^, and metal coloring^20^. The unique optical and electronic properties of PCs make them ideal platforms for a plethora of applications since these structures can behave as an effective medium and display photonic stopbands (PSBs) in different regions of the spectrum to control light–matter interactions at the nanoscale with precision^21^.

‘Hybrid’ PC structures can be defined as the combination of several individual PCs within a single complex PC structure, the spectrum of which corresponds to the combination of the spectra of each individual PC composing the hybrid structure. Hybrid PCs allow the generation of advanced systems to control light across the spectral regions with versatility, featuring different PSBs with flexible characteristics (e.g. combination of narrow and broad PSBs) for specific applications such as light filtering, sensing, etc. PCs can be synthesized by multiple fabrication methods, including lithography and dry etching^22–24^, vertical selective oxidation^25^, fibre-pulling^26^, embossing^27^, and self-organization^28,29^. However, the generation of hybrid PCs by these techniques remains challenging. Among alternative fabrication methods, anodization–electrochemical oxidation of metals–has been recently demonstrated as a suitable technique to fabricate complex metal oxide nanoporous PC structures since this cost-competitive, precise, and industrially scalable method enables the generation of PCs with high degree of regularity, flexibility, optimal resolution, and high aspect ratio^30,31^.

The intrinsic relationship between the fabrication parameters and the nanoporous structure of the resulting anodic oxide films enables the synthesis of multiple PC structures, including optical microcavities^32^, Fabry-Pérot interferometers^33^ bandpass filters^34^, distributed Bragg reflectors^20,35^, and gradient-index filters^36^. Of all the nanoporous anodic oxides produced by anodization of metals, nanoporous anodic alumina (NAA) produced by anodization of aluminum offers a unique platform material to generate complex PCs with versatile optical properties due to its excellent physical and chemical properties, highly controllable and flexible pore geometry, chemical resistance, thermal stability, mechanical robustness, non-cytotoxicity, and optoelectronic properties^37^. However, the generation of complex NAA-PCs poses some challenges due to the presence of the oxide barrier layer at the bottom tip of the nanopores, which prevents the diffusion of ionic species (e.g. Al^3+^, O^2−^, OH^−^, etc.) from and to the bulk electrolyte and regulates the transfer of electrons from anode to cathode during anodization^38–42^. Although pioneering studies demonstrated that this inherent drawback can be overcome through pulse-like anodization approaches switching the regime between mild and hard conditions^43–47^, the low controllability of hard anodization (i.e. fast oxide growth rate, heat generation, low porosity level, etc.) has limited the applicability of this approach to synthesize complex NAA-PCs^38^.

Recently, some studies by us and others have demonstrated that pulse-like anodization under mild conditions enables a more controllable approach to generate a wide range of NAA-PCs (e.g. distributed Bragg reflectors (DBR)^20^, optical microcavities^32^, gradient-index filters (GIFs)^36^, and apodized gradient-index filters (APO-GIFs)^48^). The utilization of rationally designed anodization profiles (i.e. apodized-sinusoidal, pseudo-sinusoidal, sinusoidal, sawtooth-like, and pseudo-stepwise) under suitable anodization conditions makes it possible to directly translate anodizing current density or voltage pulses into porosity modulations with precision, overcoming the inherent limitations of anodization for the in-depth engineering of the NAA’s PSB. In particular, we have recently proved that stacked NAA-bandpass filters, which consist of layers of NAA produced with different pseudo-stepwise pulse anodization periods, lead to the generation of NAA-PCs with unprecedented broad transmission bands across the UV-visible-NIR spectrum^34^. We also envisage this nanofabrication concept for the generation of hybrid nanoporous anodic alumina photonic crystals (Hy-NAA-PCs) with unique optical properties.

**Figure 1:**
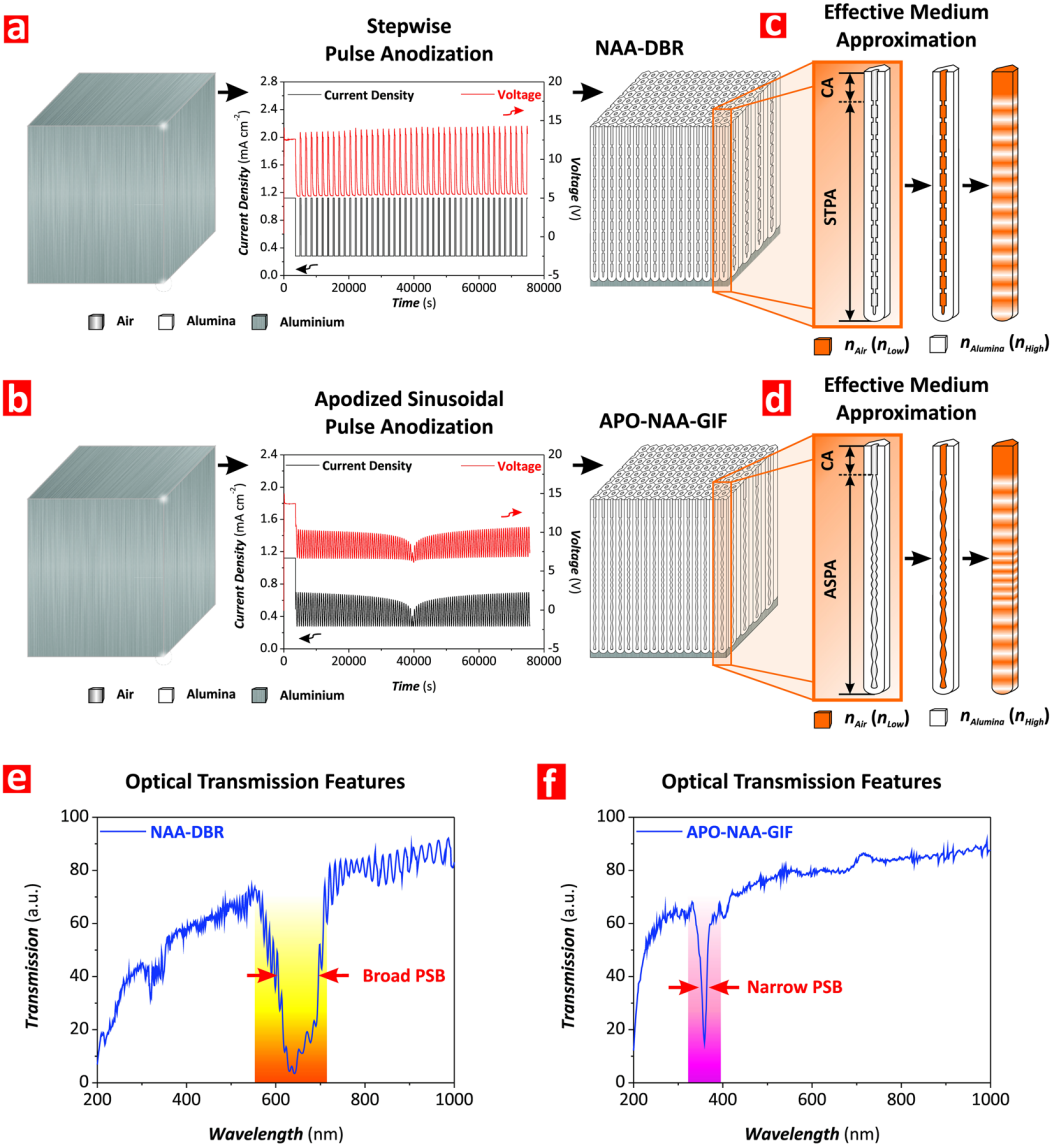
Fabrication of NAA-DBRs and APO-NAA-GIFs by stepwise pulse anodization (STPA) and apodized sinusoidal pulse anodization (ASPA), respectively. (**a,b**) Schematics illustrating the STPA and ASPA processes, representative anodization profiles, and nanoporous structure of NAA-DBRs and APO-NAA-GIFs, respectively. (**c,d**) Graphical correlation between nanopore geometry and distribution of refractive index in the structure of NAA-DBRs and APO-NAA-GIFs, respectively (Note: *n*_*Air*_ (*n*_*Low*_) and *n*_*Alumina*_ (*n*_*High*_) are the refractive indices of air and alumina). (**e,f**) Representative transmission spectra showing the features of the photonic stopband (PSB) of NAA-DBRs and APO-NAA-GIFs, respectively.

Herein, we present a novel nanofabrication approach that enables the production of Hy-NAA-PCs composed of different combinations of DBRs and APO-GIFs in a single PC structure. This one-step anodization method (‘heterogeneous pulse anodization’–HPA) is a combination of logarithmic negative apodized sinusoidal and stepwise pulse anodizations under mild conditions to attain a better controllability over the porosity and growth rate of the anodic oxide (Fig. 1). We systematically assess the effects of the fabrication parameters (i.e. anodization period, relative and total anodization time, pore widening time, and arrangement of PC structures) over the optical properties of Hy-NAA-PCs, including the position and bandwidth of the characteristic PSBs and interferometric colors. These Hy-NAA-PCs are fabricated with either two or three individual PCs featuring different geometries, demonstrating that the number, the position, and the bandwidth of the characteristic PSBs for each PC embedded within the hybrid PC structures can be precisely engineered to produce a set of unique Hy-NAA-PCs with outstanding optical properties.

## Results and Discussion

### Fabrication and Structural Characterization of Hy-NAA-PCs

Figure 1 displays schematic representations of the structure of NAA-DBRs and APO-NAA-GIFs, which are the basic unit PC structures used in our study to create Hy-NAA-PCs by HPA. As these reveal, NAA-DBRs feature a stepwise modulation of the pore diameter in depth that follows with precision the stepwise current density profile applied during the stepwise pulse anodization (STPA) process (Fig. 1a). In the case of APO-NAA-GIFs, the nanoporous structure is composed of stacked layers of NAA featuring a sinusoidally modified porosity in depth produced during apodized sinusoidal pulse anodization (ASPA), where the higher the anodization current density the higher the level of porosity following a logarithmic negative apodization function with time-dependent current density amplitude (Fig. 1b). These fabrication methods allow the precise engineering of the effective medium of NAA in depth to tune the features of the PSB (i.e. position of central wavelength and full width at half maximum) across the spectral regions with precision, as demonstrated in previous studies (Fig. 1c,d)^20,48^. Figure 1e,f show representative transmission spectra of NAA-DBRs and APO-NAA-GIFs, where the former shows a much broader characteristic PSB than that of the latter PC, which displays a characteristically well-resolved and narrow PSB. As demonstrated in previous studies, the position of the characteristic PSB (*λ*_*PSB*_) of these PCs can be precisely positioned across the spectral regions, from UV to IR, by modifying the anodization period (i.e. time between consecutive anodization pulses–*T*_*P*_)^20,48^. Figure S1 (Supporting Information) shows the dependency of *λ*_*PSB*_ with *T*_*P*_ for NAA-DBRs and APO-NAA-GIFs, which was found to follow a linear trend in both cases. Although *λ*_*PSB*_ is red-shifted with *T*_*P*_ for NAA-DBRs and APO-NAA-GIFs, the former type of NAA-PC shows a slightly stronger dependency with *T*_*P*_ as denoted by the slopes of the fitting lines (i.e. 0.62 ± 0.02 and 0.42 ± 0.01 nm s^−1^ for NAA-DBRs and APO-NAA-GIFs, respectively). Another interesting observation is that at *T*_*P*_ > 1150 s, the position of the PSB of NAA-DBRs is at longer wavelengths than that of their APO-NAA-GIFs counterparts produced with the same anodization period. This trend is opposite at *T*_*P*_ > 1150 s, where APO-NAA-GIFs feature *λ*_*PSB*_ at longer wavelengths than that of NAA-DBRs for any *T*_*P*_.

**Figure 2:**
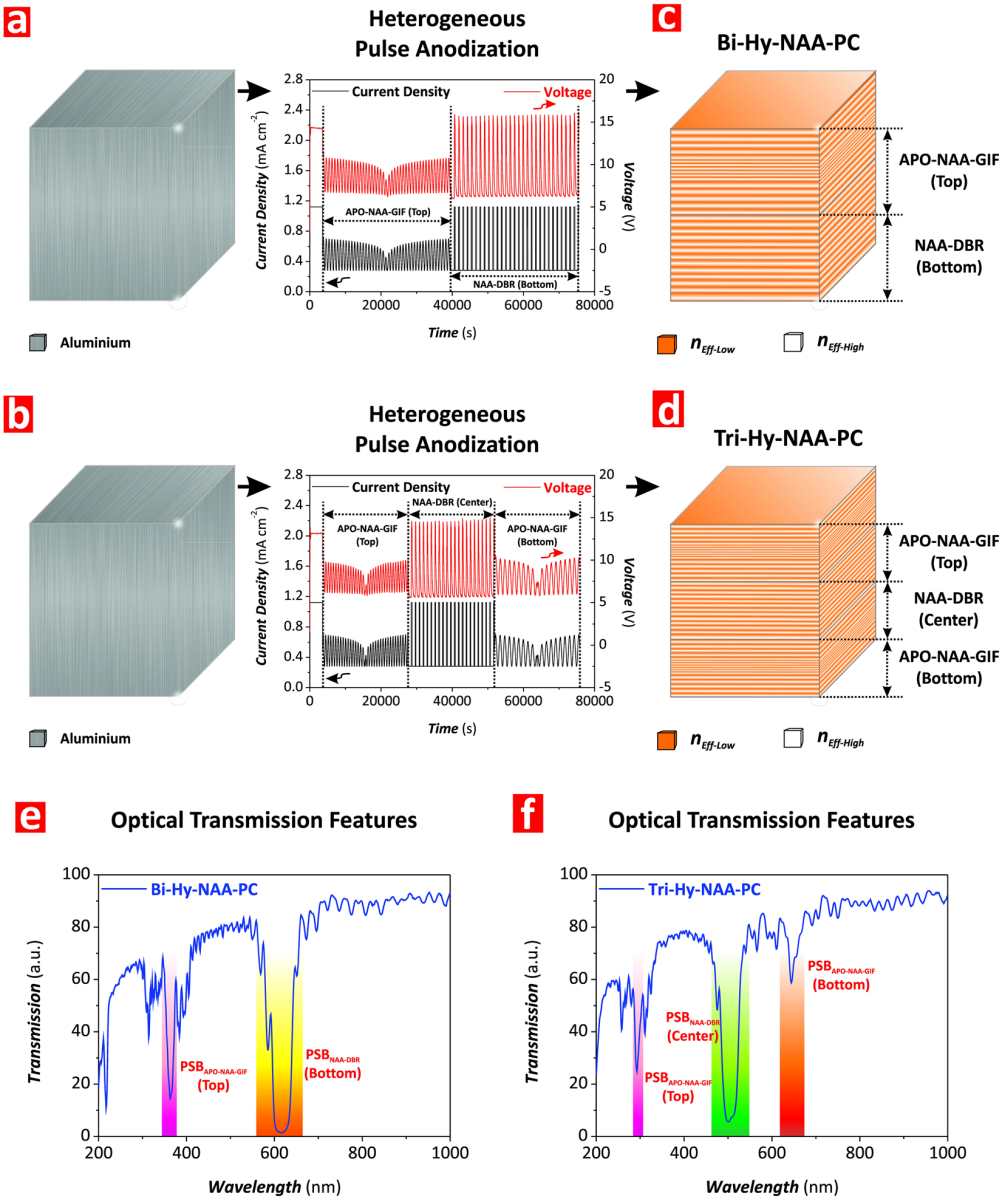
Fabrication of Bi- and Tri-Hy-NAA-PCs by heterogeneous pulse anodization (HPA). (**a,b**) Schematics illustrating the HPA processes used to produce Bi- and Tri-Hy-NAA-PCs with representative anodization profiles, respectively. (**c,d**) Schematic illustration of the distribution of effective refractive index in depth for Bi- and Tri-Hy-NAA-PCs, respectively (Note: *n*_*Eff-Low*_ and *n*_*Eff-High*_ are the low and high effective refractive indices, respectively). (**e**,**f**) Representative transmission spectra showing the features of the photonic stopbands (PSBs) of NAA-DBRs and APO-NAA-GIFs embedded within the structure of Bi- and Tri-Hy-NAA-PCs, respectively.

Figure 2a displays a conceptual illustration of Hy-NAA-PCs, where NAA-DBRs and APO-NAA-GIFs are combined into the same PC structure by HPA process. In this versatile top-down nanofabrication approach, the anodization profile can be modified to exchange the arrangement of the individual PC structures within the Hy-NAA-PC at will as well as to modify the number and characteristics of these PCs to control light in a versatile fashion for specific applications. In our study, we developed and assessed two types of Hy-NAA-PCs: i) Bi-Hy-NAA-PCs, composed of two individual NAA-PCs (Fig. 2a), and ii) Tri-Hy-NAA-PCs, formed by the generation of three individual NAA-PCs (Fig. 2b). As Fig. 2c,d show, the transmission spectrum of Hy-NAA-PCs can be approximated to the resulting combination of the spectra of each individual NAA-PCs embedded within the hybrid PC structure.

**Figure 3:**
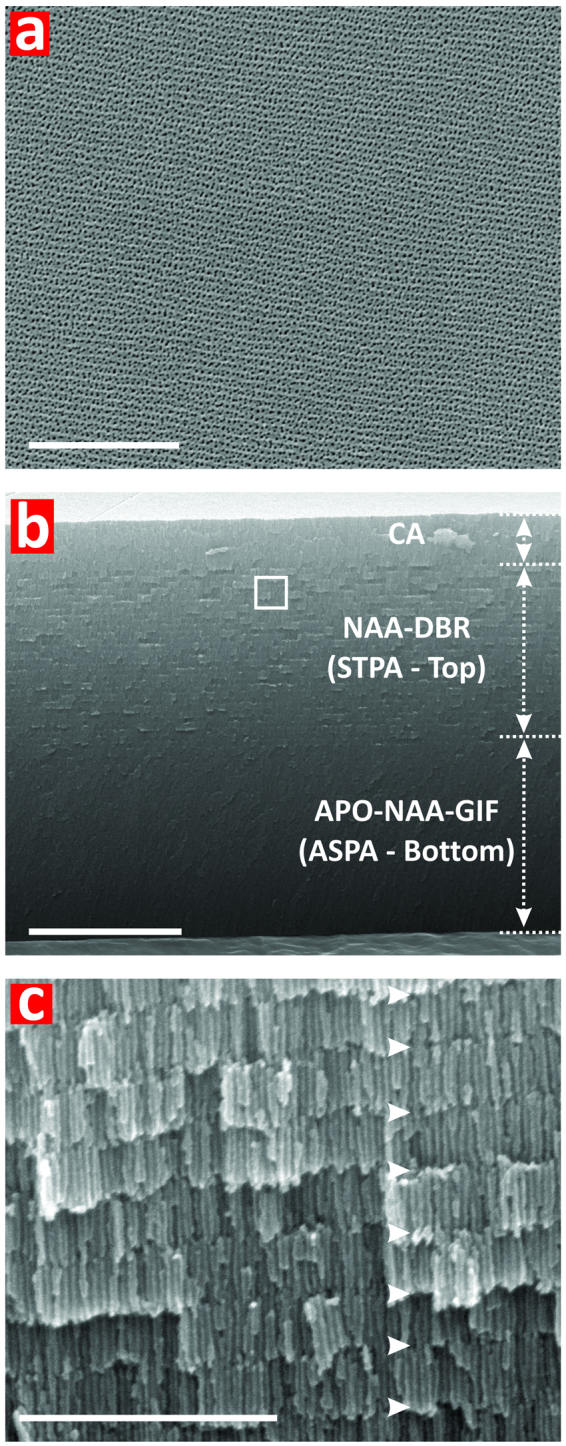
Structural characterization of Bi- and Tri-Hy-NAA-PCs produced by heterogeneous pulse anodization (HPA). (**a**) Top FEG-SEM image of a Bi-Hy-NAA-PC combining one NAA-DBR on its top and one APO-NAA-GIF on its bottom produced by HPA and *t*_*pw*_ = 6 min (scale bar = 1 μm). (**b**) Cross-sectional FEG-SEM image showing the different parts of the Bi-Hy-NAA-PC (Note: CA = constant anodization) (scale bar = 5 μm). (**c**) Magnified view of white rectangle shown in (**b**) revealing the modulation of pore diameter in depth, where white arrowheads denote the period length (i.e. distance between consecutive pulses in the structure of the Bi-Hy-NAA-PC) (scale bar = 1 μm).

Figure 3 compiles a set of representative FEG-SEM images illustrating the structure of Hy-NAA-PCs. As these images reveal, Hy-NAA-PCs feature a random distribution of nanopores across their top surface, which have an as-produced diameter (*d*_*P*_) of 10 ± 2 nm (i.e. *t*_*pw*_ = 0 min) and that is enlarged up to 20 ± 3 nm after 6 min of pore widening treatment (Fig. 3a). Cross-sectional FEG-SEM images demonstrate that the nanopore diameter in Hy-NAA-PCs is modulated in depth following the HPA current density profile (Fig. 3b,c), where each individual PC structure can be discerned.

### Effect of Structural Arrangement on the Optical Properties of Bi-Hy-NAA-PCs

The structure of Bi-Hy-NAA-PCs is composed of two individual NAA-PCs: one NAA-DBR and one APO-NAA-GIF, in which these can be alternately arranged as the top or bottom structures (Fig. 2a). First, we fabricated two types of Bi-Hy-NAA-PCs featuring two configurations to study the effect of the arrangement of the NAA-DBR and APO-NAA-GIF on the optical properties of Bi-Hy-NAA-PCs and assess the capabilities of our HPA approach (Table 1): i) Bi-Hy-NAA-PCs, where the APO-NAA-GIF is located at the top of the hybrid NAA-PC structure and the NAA-DBR is located at its bottom, and ii) Inv-Bi-Hy-NAA-PCs with an inverted configuration, in which the NAA-DBR is located at the top and the APO-NAA-GIF at the bottom of the PC structure.

**Table 1:** Summary table compiling the structural arrangement and fabrication conditions for each Bi-Hy-NAA-PC structure assessed in this study.

Type	Top PC Structure *t*_*An-Top*_ (h)–*T*_*P*_ (s)	Bottom PC Structure *t*_*An-Bottom*_ (h)–*T*_*P*_ (s)	*t*_*An*_ (h)
Bi-Hy-NAA-PC	APO-NAA-GIF 10 h–800 s	NAA-DBR 10 h–1200 s	20 h
Inv-Bi-Hy-NAA-PC	NAA-DBR 10 h–1200 s	APO-NAA-GIF 10 h–800 s	20 h
Bi-Hy-NAA-PC	APO-NAA-GIF 7.5 h–800 s	NAA-DBR 7.5 h–1200 s	15 h
Bi-Hy-NAA-PC	APO-NAA-GIF 5 h–800 s	NAA-DBR5 h–1200 s	10 h

**Figure 4:**
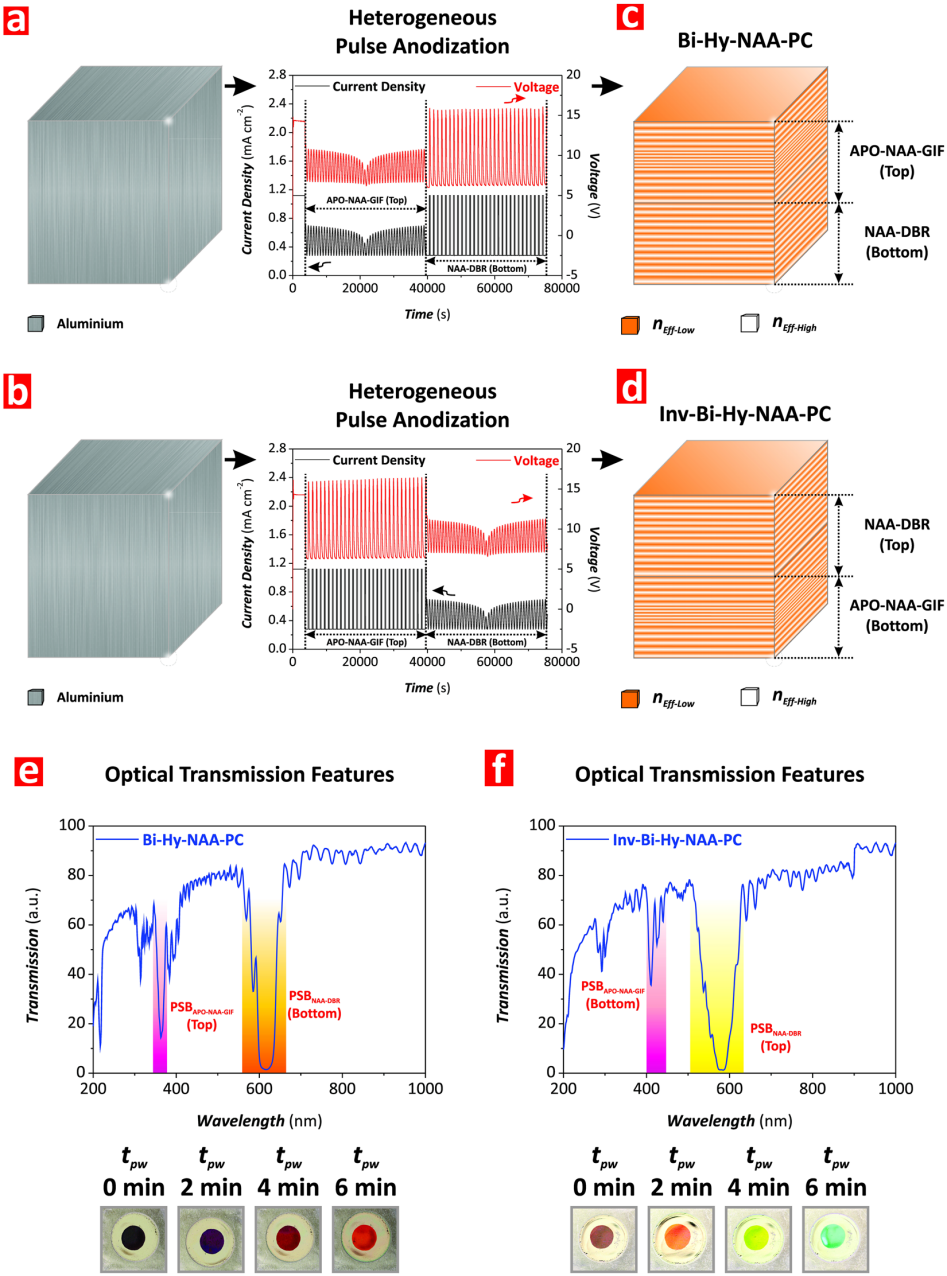
Fabrication of Bi- and Inv-Bi-Hy-NAA-PCs by heterogeneous pulse anodization (HPA). (**a,b**) Schematics illustrating the HPA processes used to produce Bi- and Inv-Bi-Hy-NAA-PCs with representative anodization profiles, respectively. (**c,d**) Schematic illustration of the distribution of effective refractive index in depth for Bi- and Inv-Bi-Hy-NAA-PCs, respectively. (**e,f**) Representative transmission spectra showing the features of the photonic stopbands (PSBs) of NAA-DBRs and APO-NAA-GIFs embedded within the structure of Bi- and Inv-Bi-Hy-NAA-PCs and digital pictures showing the interferometric color displayed by these PC structures as a function of *t*_*pw*_, respectively.

Figure 4a,b display representative anodization profiles used to produce the Bi-Hy-NAA-PCs and Inv-Bi-Hy-NAA-PCs analyzed in our study. NAA-DBRs were fabricated with *T*_*P*_ = 1200 s (i.e. *t*_*min*_ = 960 s and *t*_*max*_ = 240 s) and 30 pulses (i.e. relative anodization time = 10 h), and APO-NAA-GIFs with *T*_*P*_ = 800 s, *J*_*Offset*_ = 0.280 mA cm^−2^, *ΔA*_*J*_ = 0.210 mA cm^−2^ and 45 pulses (i.e. relative anodization time = 10 h). Note that to avoid overlapping of PSBs, using Figure S1 as a reference, the fabrication conditions were selected so the PSB of NAA-DBRs and APO-NAA-GIFs were located at ~700 nm and ~450 nm, respectively.

The relative (i.e. time or number of pulses used to produce each individual PC structure) and total anodization times were fixed to 10 and 20 h, respectively, in both types of Hy-NAA-PCs. HPA is a continuous top-down fabrication process controlled by the diffusion of ionic species (e.g. Al^3+^, O^2−^, OH^−^) along the nanopores and the oxide barrier layer. However, the anodization conditions used in our study make it possible the direct translation of current density pulses (input) into porosity changes in depth, as denoted by the anodization voltage (output), enabling the precise and versatile engineering of the nanoporous structure of NAA (Fig. 4c,d).

Figure 4e,f show representative transmission spectra of Bi-Hy-NAA-PCs and Inv-Bi-Hy-NAA-PCs fabricated with the above-mentioned characteristics as a function of the pore widening time (*t*_*pw*_). In both cases, these NAA-PCs display two types of PSB at *t*_*pw*_ = 0 min, one broad PSB with *λ*_*PSB*_ at ~700 nm and one narrow PSB with *λ*_*PSB*_ at ~450 nm, which correspond to the NAA-DBR (PSB_NAA-DBR_) and the APO-NAA-GIF PC structures (PSB_APO-NAA-GIF_), respectively. It is observed that the position of the two characteristic PSBs undergoes a blue shift with increasing *t*_*pw*_, which is in good agreement with previous studies reported elsewhere^48,49^. For instance, at *t*_*pw*_ = 0 min, *λ*_*PSB*_ for the APO-NAA-GIF structure in Bi-Hy-NAA-PCs and Inv-Bi-NAA-PCs was 449 ± 1 and 452 ± 1 nm, respectively, which is certainly close to the value estimated from Figure S1 (~450 nm). This observation is confirmed for the NAA-DBR structure too, which central wavelength was positioned at 692 ± 1 and 660 ± 1 nm in Bi-Hy-NAA-PCs and Inv-Bi-NAA-PCs, respectively (estimated value ~700 nm). These observations demonstrate that the position of the PSB_NAA-DBR_ and PSB_APO-NAA-GIF_ is practically independent on the arrangement of these PCs within the structure of Hy-NAA-PCs during the HPA process but highly dependent on the anodization period (NAA-DBR–*T*_*P*_ = 1200 s and APO-NAA-GIF–*T*_*P*_ = 800 s).

As these graphs reveal, the position of PSB_NAA-DBR_ and PSB_APO-NAA-GIF_ in Bi-Hy-NAA-PCs and Inv-Bi-NAA-PCs can be linearly tuned by a pore widening treatment. Linear fittings showing the dependence of *λ*_*PSB*_ with *t*_*pw*_ for each individual PC structure reveal that in Bi-Hy-NAA-PCs *λ*_*PSB*_ is blue-shifted with *t*_*pw*_ at similar rates (i.e. −14 ± 1 and −13 ± 2 nm min^−1^ for PSB_APO-NAA-GIF_ and PSB_NAA-DBR_, respectively). However, in the case of Inv-Bi-Hy-NAA-PCs, the position of the central wavelength of the NAA-DBR located at the top part was observed to change at slightly higher rate than its non-inverted counterpart (i.e. −22 ± 1 and −11 ± 2 nm min^−1^ for PSB_NAA-DBR_ and PSB_APO-NAA-GIF_, respectively) (Figure S2–Supporting Information). The analysis of the full width at half maximum (FWHM) of the PSB_NAA-DBR_ and PSB_APO-NAA-GIF_ for Bi-Hy-NAA-PCs and Inv-Bi-Hy-NAA-PCs reveals that, as expected, the FWHM of the PSB of NAA-DBRs are fairly wider than that of the APO-NAA-GIFs. Furthermore, in general, it is observed that the transmission bands are broader for Inv-Bi-Hy-NAA-PCs than those of Bi-Hy-NAA-PCs with increasing *t*_*pw*_. For example, in the case of Bi-Hy-NAA-PCs, the FWHM_APO-NAA-GIF_ and FWHM_NAA-DBR_ for *t*_*pw*_ 0 and 6 min were measured to be 8 ± 1 and 18 ± 1 nm, and 20 ± 1 and 54 ± 1 nm, respectively. However, for Inv-Bi-Hy-NAA-PCs, the values of FWHM_APO-NAA-GIF_ and FWHM_NAA-DBR_ for *t*_*pw*_ 0 and 6 min were 10 ± 1 and 29 ± 1 nm, and 30 ± 1 and 160 ± 1 nm, respectively.

The arrangement of individual PC structures from Bi-Hy-NAA-PCs to Inv-Bi-Hy-NAA-PCs produced an increment of FWHM of 23 and 49% for the APO-NAA-GIF at *t*_*pw*_ = 0 and 6 min, and 34 and 66% for the NAA-DBR. Therefore, our analysis reveals that, while the interchange of individual PCs within the structure of Bi-Hy-NAA-PCs has not significant effect on the position of the PSBs, it significantly affects the FWHM of the PSBs.

Figure 4e,f compile digital images of Bi-Hy-NAA-PCs and Inv-Bi-Hy-NAA-PCs as a function of *t*_*pw*_, showing that these PCs display vivid interferometric colors across the UV-visible-NIR spectrum. In good agreement with our observations about the effect of the arrangement of individual PC structures on the *λ*_*PSB*_ and FWHM of Hy-NAA-PCs, it is observed that the interferometric color at a fixed *t*_*pw*_ is slightly blue shifted in Inv-Bi-Hy-NAA-PCs as compared to their reference Bi-Hy-NAA-PCs (e.g. green and orange colors for the former and latter at *t*_*pw*_ = 6 min, respectively). This result can be ascribed to the broader and more intense PSB of the NAA-DBR embedded in the structure of Inv-Bi-Hy-NAA-PCs, which is the main contributor to the efficient reflection of light (i.e. color) from Bi-Hy-NAA-PCs.

### Effect of Relative and Total Anodization Times on the Optical Properties of Bi-Hy-NAA-PCs

As mentioned above, the structure of Bi-Hy-NAA-PCs is composed of two individual PCs, with one APO-NAA-GIF and one NAA-DBR located at the top and bottom of the hybrid photonic structure, respectively. As shown in the previous section, these NAA-PCs were fabricated at fixed relative anodization time (*t*_*An*_) or number of pulses and anodization period, which were set at *t*_*An-Top*_ = 10 h (or 45 pulses) and *T*_*P*_ = 800 s for the APO-NAA-GIF, and at *t*_*An-Bottom*_ = 10 h (or 30 pulses) and *T*_*P*_ = 1200 s for the NAA-DBR, respectively. HPA is a versatile nanofabrication approach that can be used to engineer the characteristic features of each individual PC within Hy-NAA-PCs. To gain insight into the capabilities of HPA, we assessed the effect of the relative anodization times (i.e. *t*_*An-Top*_ and *t*_*An-Bottom*_) and the total anodization time (*t*_*An*_) on the optical properties (i.e. *λ*_*PSB*_, FWHM, and interferometric color) of Bi-Hy-NAA-PCs by systematic modification of these fabrication parameters. Table 1 summarizes the set of *t*_*An-Top*_, *t*_*An-Bottom*_, and *t*_*An*_ values used in our study, the transmission spectra of which are shown in Figure S3 (Supporting Information).

**Figure 5:**
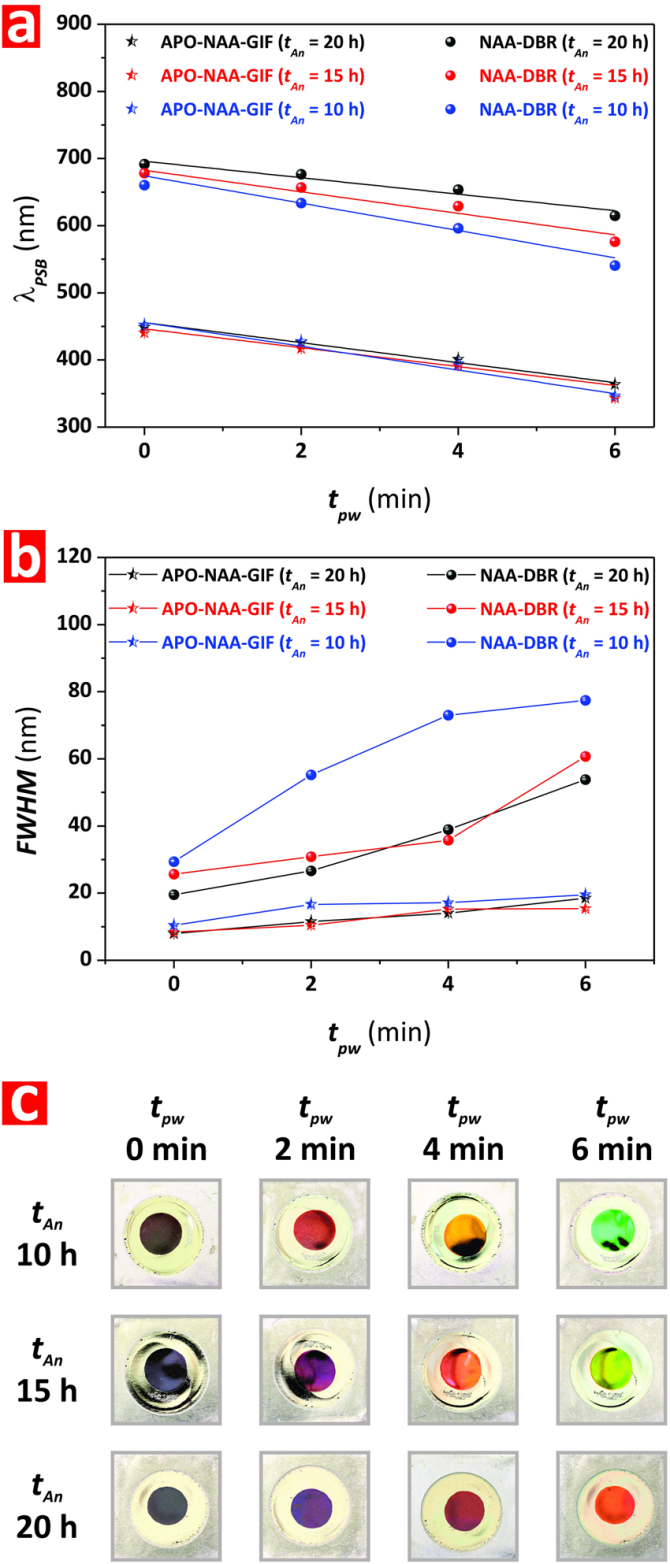
Effect of total anodization time (*t*_*An*_) and pore widening time (*t*_*pw*_) on the optical features (position of central wavelength–*λ*_*PSB*_, full-width at half maximum–FWHM, and interferometric color) of Bi-Hy-NAA-PCs. (**a**) Effect of *t*_*An*_ and *t*_*pw*_ on *λ*_*PSB*_. (**b**) Effect of *t*_*An*_ and *t*_*pw*_ on FWHM. (**c**) Effect of *t*_*An*_ and *t*_*pw*_ on interferometric color.

Figure 5 shows the effect of *t*_*An*_ (from 10 to 20 h) on the *λ*_*PSB*_, FWHM, and interferometric color of the Bi-Hy-NAA-PCs produced at *t*_*An-Top*_ = *t*_*An-Bottom*_ (from 5 to 10 h) and different pore widening times (from 0 to 6 min). Figure 5a displays the dependency of *λ*_*PSB*_ for the APO-NAA-GIF and NAA-DBR in the structure of Bi-Hy-NAA-PCs as a function of *t*_*An*_ and *t*_*pw*_. It is observed that, in the case of the NAA-DBR structure, *λ*_*PSB*_ undergoes a blue shift as *t*_*An*_ is reduced from 20 to 10 h (i.e. *t*_*An-Top*_ = *t*_*An-Bottom*_ = 10 and 5 h, respectively).

This blue shift is observed to be more significant as *t*_*pw*_ increases from 0 to 6 min. However, this trend is not observable in the case of the APO-NAA-GIF structure, which shows no statistically significant changes in *λ*_*PSB*_ with *t*_*An*_. In good agreement with previous results, *λ*_*PSB*_ undergoes a blue shift with *t*_*pw*_, which was found to be statistically the same at any *t*_*An*_. Figure 5b shows the dependency of the FWHM of the PSB_APO-NAA-GIF_ and PSB_NAA-DBR_ structures in Bi-Hy-NAA-PCs as a function of *t*_*An*_ and *t*_*pw*_. At first glance, it is verified that the longer *t*_*pw*_ the wider FWHM in both the APO-NAA-GIF and NAA-DBR structure, although it is worth noting that this effect is much more significant for the latter individual PC structure. Another aspect to note is that the FWHM shows similar behavior for both PCs at *t*_*An*_ = 15 and 20 h. However, a reduction of *t*_*An*_ down to 10 h results in a significant enlargement of the FWHM of the PSB_NAA-DBR_. This increment is also observed in the case of the APO-NAA-GIF, although in a less significant manner than that of its NAA-DBR counterpart.

As far as the interferometric color of these Bi-Hy-NAA-PCs is concerned, Fig. 5c compiles a set of digital pictures of these NAA-based PCs, in which it is observed that a reduction of the total anodization time results in a slight blue shift in the interferometric color displayed by these PCs, which is in good agreement with our analysis performed in Fig. 5a. For instance, at *t*_*pw*_ = 0 min all the samples are transparent, indicating that the PSB is located in the NIR region of the spectrum. At *t*_*pw*_ = 2 min, those Bi-Hy-NAA-PCs produced at *t*_*An*_ = 10 h display red color, while those produced at 15 and 20 h are still transparent. At *t*_*pw*_ = 4 min, Bi-Hy-NAA-PCs produced at *t*_*An*_ = 10, 15, and 20 h display gold, orange-red, and red color, respectively, denoting a slight blue shift in color as a result of the anodization time.

To gain further insights into the effect of the relative anodization time (i.e. time or number of pulses used to fabricate each of the individual PC structures of Bi-Hy-NAA-PCs–*t*_*An-Top*_ for APO-NAA-GIF and *t*_*An-Bottom*_ for NAA-DBR), we modified these parameters systematically while keeping the total anodization time (*t*_*An*_) constant. We assessed four Bi-Hy-NAA-PCs produced with the following values of *t*_*An-Top*_, *t*_*An-Bottom*_, and *t*_*An*_: i) *t*_*An-Top*_ = 15 h, *t*_*An-Bottom*_ = 5 h, and *t*_*An*_ = 20 h; ii) *t*_*An-Top*_ = 5 h, *t*_*An-Bottom*_ = 15 h, and *t*_*An*_ = 20 h; iii) *t*_*An-Top*_ = 5 h, *t*_*An-Bottom*_ = 10 h, and *t*_*An*_ = 15 h; and iv) *t*_*An-Top*_ = 10 h, *t*_*An-Bottom*_ = 5 h, and *t*_*An*_ = 15 h.

**Figure 6:**
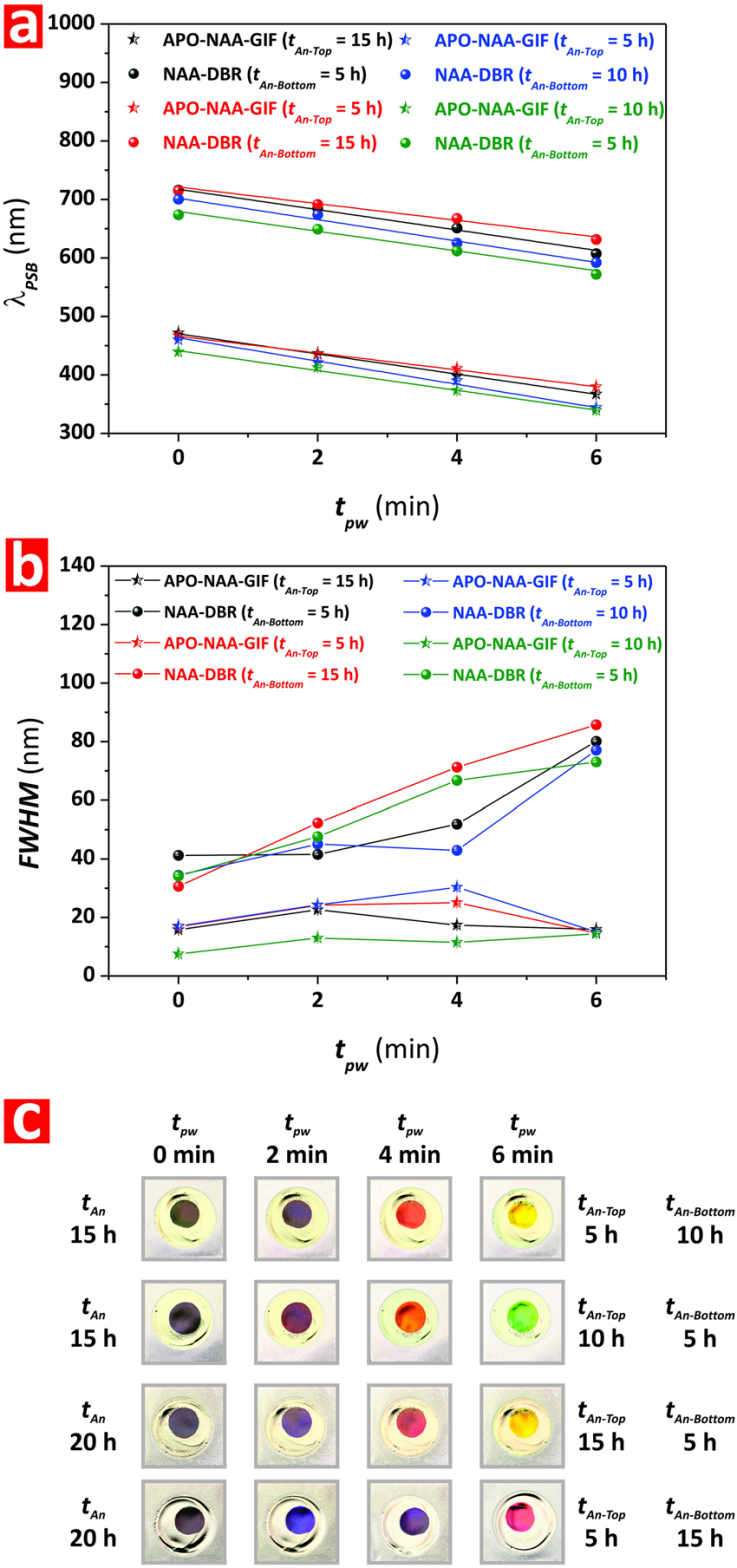
Effect of relative anodization time (*t*_*An-Top*_ and *t*_*An-Bottom*_) and pore widening time (*t*_*pw*_) on the optical features (position of central wavelength–*λ*_*PSB*_, full-width at half maximum–FWHM, and interferometric color) of Bi-Hy-NAA-PCs. (**a**) Effect of *t*_*An-Top*_, *t*_*An-Bottom*_, and *t*_*pw*_ on *λ*_*PSB*_. (**b**) Effect of *t*_*An-Top*_, *t*_*An-Bottom*_, and *t*_*pw*_ on FWHM. (**c**) Effect of *t*_*An-Top*_, *t*_*An-Bottom*_, and *t*_*pw*_ on interferometric color.

Figure 6a summarizes the effect of these fabrication parameters in combination with *t*_*pw*_ on the position of the characteristic PSB of these Bi-Hy-NAA-PCs. As a rule, it is observed that the PSB_NAA-DBR_ undergoes a slight blue shift when *t*_*An*_ is reduced from 20 to 15 h, which is further enhanced when the relative anodization time used to produce the NAA-DBR (*t*_*An-Bottom*_) is reduced from 15 to 5 h and from 10 to 5 h, and *t*_*pw*_ increased. This trend is also observed in the case of the APO-NAA-GIF structure, although the magnitude of the blue shift with both the relative and total anodization time are less significant than that observed for the NAA-DBR structure.

Figure 6b shows the effect of the relative and total anodization times combined with *t*_*pw*_ on the FWHM of the APO-NAA-GIF and NAA-DBR structures of Bi-Hy-NAA-PCs. Although it is not possible to find any consistent pattern of the effect of *t*_*An-Top*_, *t*_*An-Bottom*_, and *t*_*An*_ on the FWHM, it can be clearly observed that, likewise in previous cases, extended pore widening times lead to an enlargement of the FWHM of the PSB_NAA-DBR_. The FWHM of the PSB_APO-NAA-GIF_ is much narrower than that of its NAA-DBR counterpart at any *t*_*An-Top*_, *t*_*An-Bottom*_, *t*_*An*_, and *t*_*pw*_, and it is also confirmed that it increases with *t*_*pw*_ up to 4 min. A slight decrement of FWHM is observed from *t*_*pw*_ = 4 to 6 min, which might be associated with over etching of the APO-NAA-GIF structure.

Finally, Fig. 6c compiles a set of representative digital pictures of Bi-Hy-NAA-PCs as a function of *t*_*An-Top*_, *t*_*An-Bottom*_, *t*_*An*_, and *t*_*pw*_. The qualitative analysis of these pictures reveals that, as shown in Fig. 6a, the reduction of *t*_*An*_ leads to a slight blue shift as denoted by the interferometric color of Bi-Hy-NAA-PCs when the PSB_NAA-DBR_ is located within the visible range of the spectrum. This shift is much more evident when the relative anodization time used to produce the NAA-DBR structure (*t*_*An-Bottom*_) is reduced since, as mentioned above, the interferometric color is predominantly established by the PSB of the NAA-DBR. For example, at *t*_*An*_ = 15 h and *t*_*pw*_ = 6 min, Bi-Hy-NAA-PCs display yellow and green color at *t*_*An-Bottom*_ 10 and 5 h, respectively. This is also confirmed at *t*_*An*_ = 20 h and *t*_*pw*_ = 6 min, when Bi-Hy-NAA-PCs display red and orange-yellow color at *t*_*An-Bottom*_ 20 and 15 h, respectively.

### Effect of Structural Arrangement and Relative Anodization Time on the Optical Properties of Tri-Hy-NAA-PCs

To further demonstrate the versatility of the HPA technique to fabricate hybrid PC structures, we produced a set of Tri-Hy-NAA-PCs featuring three individual PCs in the form of APO-NAA-GIFs and NAA-DBRs. First, we analyzed the effect of the arrangement of the individual PCs on the optical properties of Tri-Hy-NAA-PCs. To this end, we fabricated two types of Tri-Hy-NAA-PCs at constant *t*_*An*_ = 20 h: Type-1) one structure composed of a NAA-DBR produced with *T*_*P*_ = 1000 s and *t*_*An-Center*_ = 6.67 h (~22 pulses–central structure) sandwiched between two APO-NAA-GIFs produced with *T*_*P*_ = 700 s and *t*_*An-Top*_ = 6.67 h (~34 pulses–top structure) and *T*_*P*_ = 1300 s and *t*_*An-Bottom*_ = 6.67 h (~18 pulses–bottom structure), and Type-2) another structure composed of an APO-NAA-GIF produced with *T*_*P*_ = 1100 s and *t*_*An-Center*_ = 6.67 h (~22 pulses–central structure) sandwiched between two NAA-DBRs produced with *T*_*P*_ = 900 s and *t*_*An-Top*_ = 6.67 h (~27 pulses–top structure), and *T*_*P*_ = 1200 s and *t*_*An-Bottom*_ = 6.67 h (~20 pulses–bottom structure).

**Figure 7:**
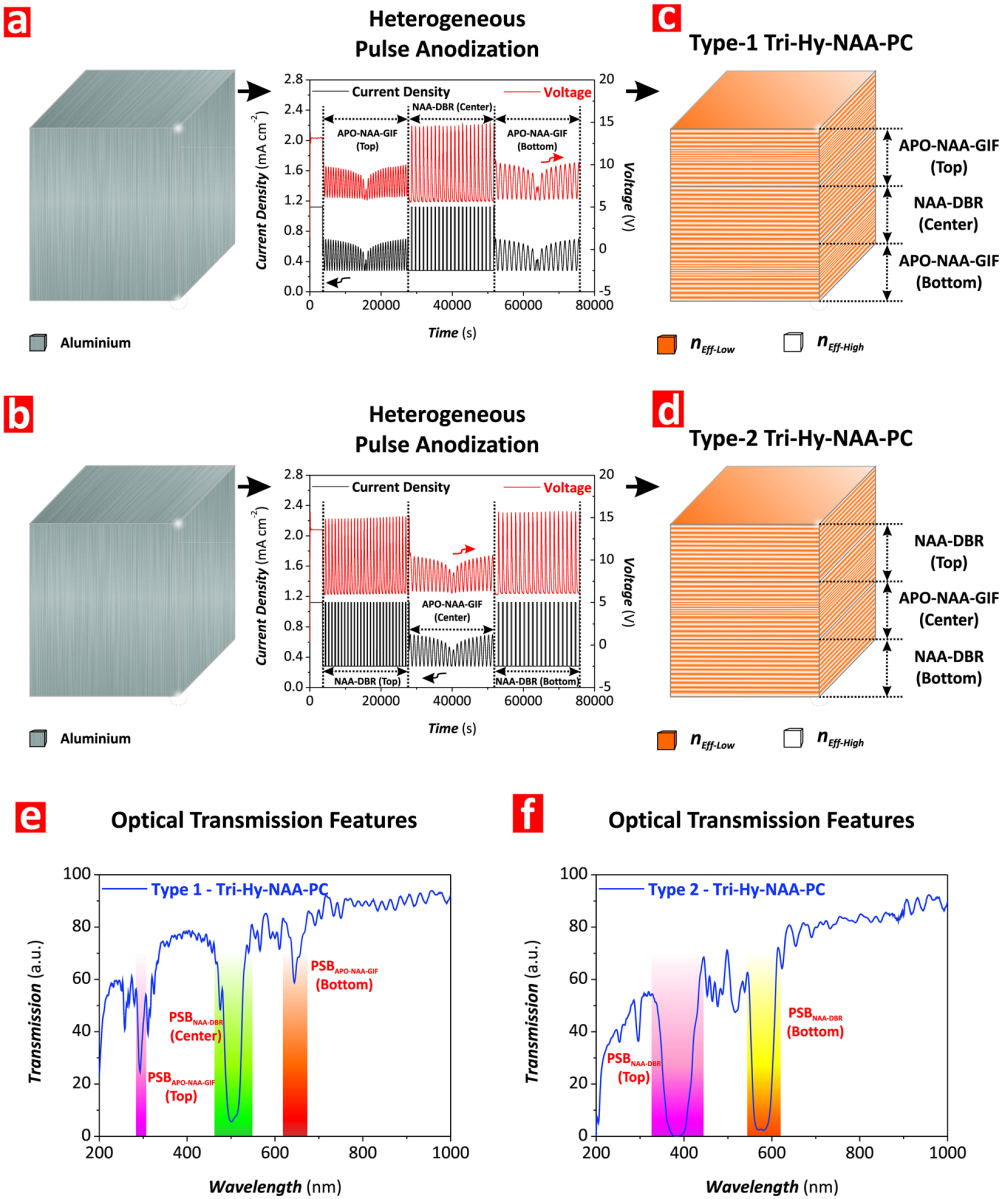
Fabrication of Type 1 and Type 2 Tri-Hy-NAA-PCs by heterogeneous pulse anodization (HPA). (**a,b**) Schematics illustrating the HPA processes used to produce Type 1 and Type 2 Tri-Hy-NAA-PCs with representative anodization profiles, respectively. (**c,d**) Schematic illustration of the distribution of effective refractive index in depth for Type 1 and Type 2 Tri-Hy-NAA-PCs, respectively. (**e,f**) Representative transmission spectra showing the features of the photonic stopbands (PSBs) of NAA-DBRs and APO-NAA-GIFs embedded within the structure of Type 1 and Type 2 Tri-Hy-NAA-PCs, respectively.

Figure 7a and b show representative anodization profiles used to produce these Tri-Hy-NAA-PCs by HPA and schematic illustrations of their structure (Fig. 7c and d). The transmission spectrum of the Type-1 hybrid PC structure displays three PSBs, the features of which are established by each individual PC structure composing the Tri-Hy-NAA-PC (Fig. 7e). However, it must be noted that the Type-2 Tri-Hy-NAA-PC structure composed of two NAA-DBRs and one APO-NAA-GIF did not show the characteristic PSB of the APO-NAA-GIF (Fig. 7f).

**Figure 8:**
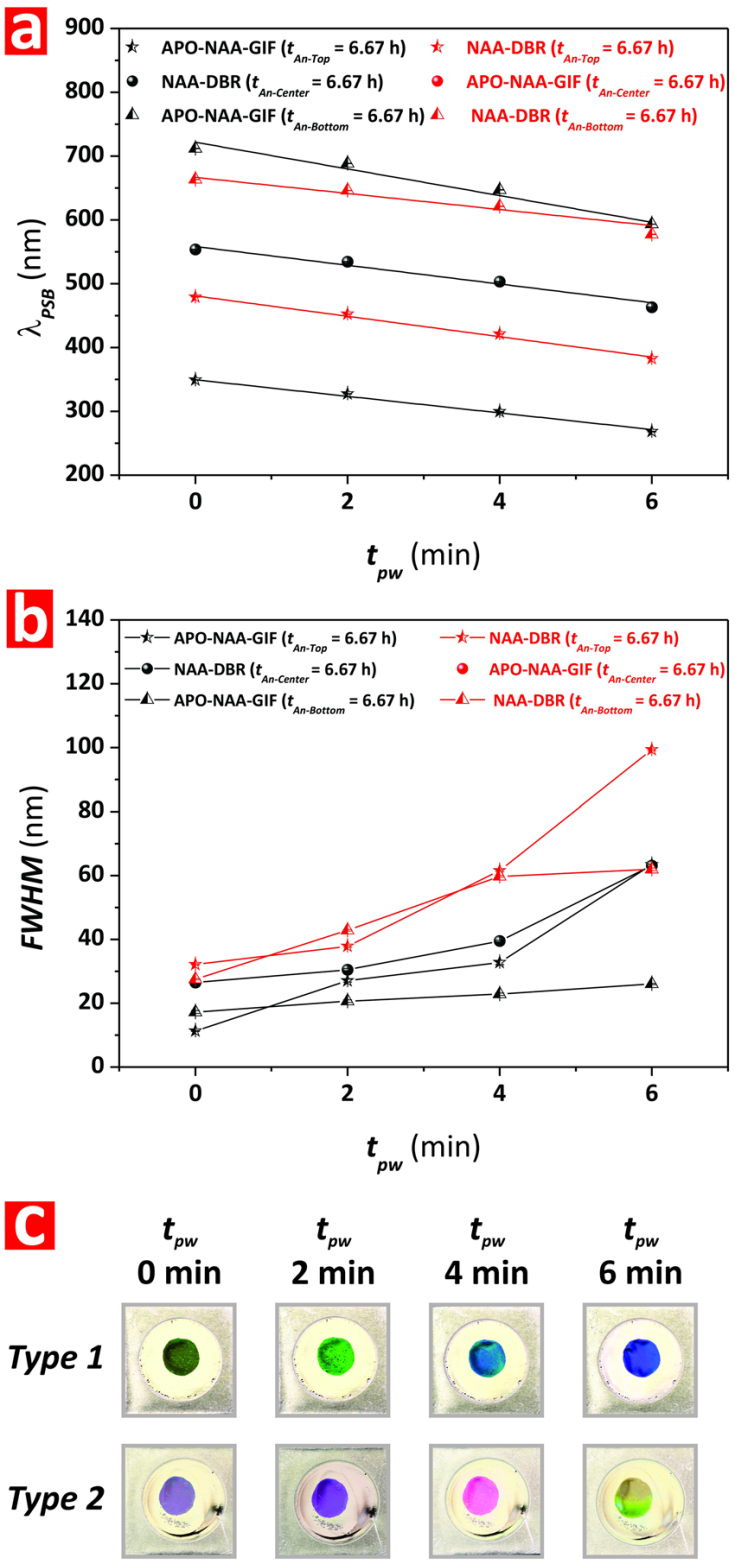
Effect of type of individual PC arrangement and pore widening time (*t*_*pw*_) on the optical features (position of central wavelength–*λ*_*PSB*_, full-width at half maximum–FWHM, and interferometric color) of Type 1 and Type 2 Tri-Hy-NAA-PCs. (**a**) Effect of structural arrangement and *t*_*pw*_ on *λ*_*PSB*_. (**b**) Effect of structural arrangement and *t*_*pw*_ on FWHM. (**c**) Effect of structural arrangement and *t*_*pw*_ on interferometric color.

Figure 8 summarizes the dependency of *λ*_*PSB*_ and FWHM of the individual PCs composing the structure of Type-1 and Type-2 Tri-Hy-NAA-PCs with *t*_*pw*_. It is clearly observed that the *λ*_*PSB*_ of these PCs embedded in the structure of Tri-Hy-NAA-PCs follows a linear trend with *t*_*pw*_, where it is blue-shifted with the pore widening process (Fig. 8a). The relative position of the three characteristic PSBs of the Type-1 Tri-Hy-NAA-PC evolves in a similar way with *t*_*pw*_, keeping an equidistant position across the spectrum according to the design of the fabrication conditions for each individual PC structure. Although the PSB of the central APO-NAA-GIF is vanished from the spectrum of the Type-2 Tri-Hy-NAA-PC, the PSBs of the top and bottom NAA-DBRs follow the same trend with *t*_*pw*_ than that observed for the Type-1. Figure 8b shows how the FWHM of these Tri-Hy-NAA-PCs varies with *t*_*pw*_. This reveals that, for the Type-1 Tri-Hy-NAA-PC structure, the FWHM of the NAA-DBRs are broader than those of the APO-NAA-GIFs, although this difference is reduced as compared to that shown in Bi-Hy-NAA-PCs. Finally, Fig. 8c compiles digital pictures of these Tri-Hy-NAA-PCs. These images reveal that, while the interferometric color displayed by the Type-1 structure is clearly established by the PSB of the central NAA-DBR, the Type-2 structure shows a complex pattern of colors, which result from the combination of the bands of the two NAA-DBRs embedded into its structure. In both cases, the interferometric color is blue-shifted with *t*_*pw*_.

As mentioned above, the PSB of the APO-NAA-GIF structure of the Type-2 Tri-Hy-NAA-PC, which was embedded between two NAA-DBRs, was found to vanish from the transmission spectrum of these PCs. To establish optimal fabrication conditions to overcome this limitation so Tri-Hy-NAA-PCs show the three characteristic PSBs in their transmission spectrum, we fabricated a set of Type-1 and Type-2 Tri-Hy-NAA-PCs by systematically modifying the relative anodization times (*t*_*An-Top*_, *t*_*An-Center*_, and *t*_*An-Bottom*_) while keeping constant the total anodization time (*t*_*An*_).

**Figure 9:**
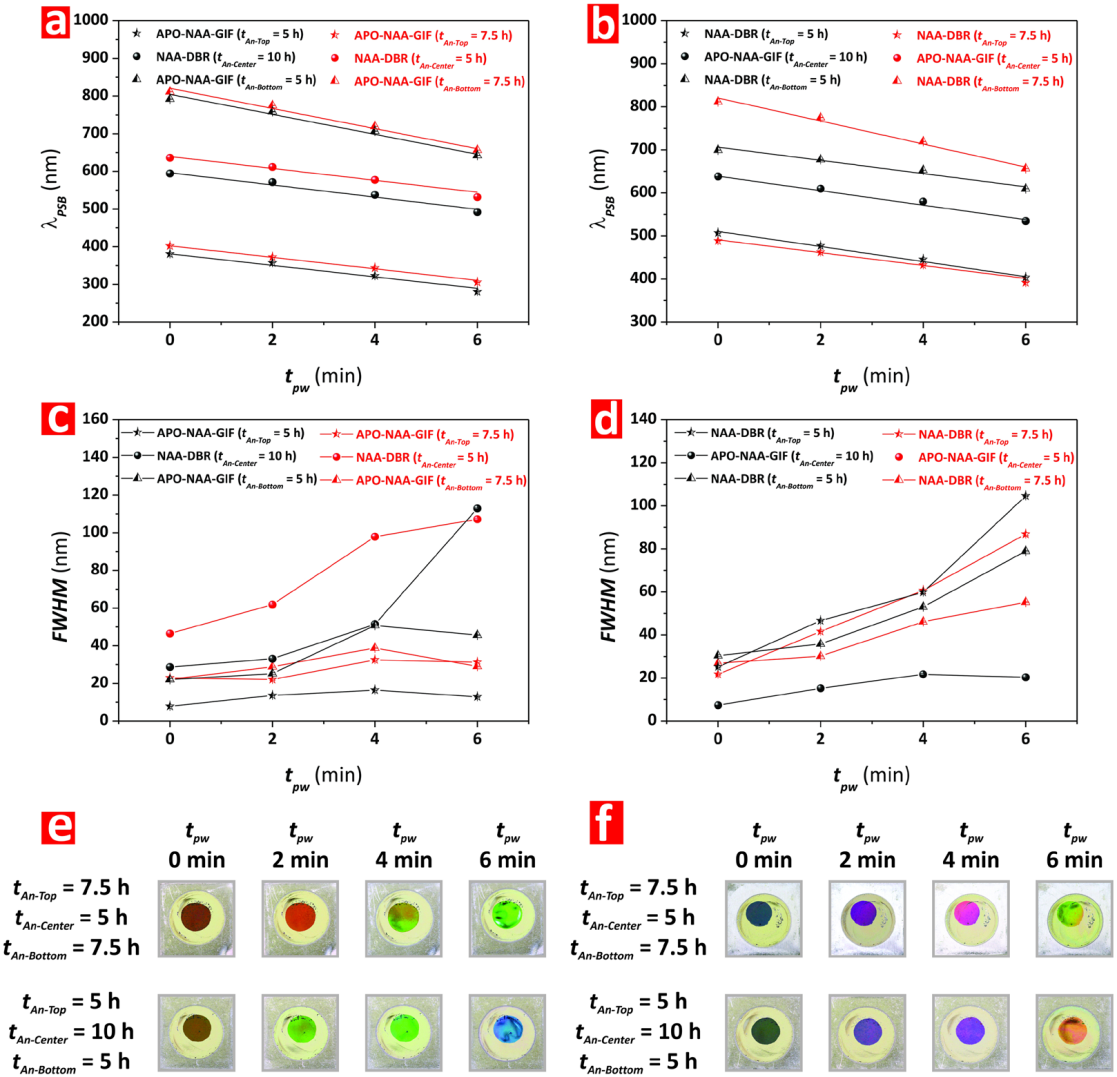
Effect of relative anodization time (*t*_*An-Top*_, *t*_*An-Center*_, and *t*_*An-Bottom*_) and pore widening time (*t*_*pw*_) on the optical features (position of central wavelength–*λ*_*PSB*_, full-width at half maximum–FWHM, and interferometric color) of Type 1 and Type 2 Tri-Hy-NAA-PCs. (**a**,**b**) Effect of *t*_*An-Top*_, *t*_*An-Bottom*_, and *t*_*pw*_ on *λ*_*PSB*_ of Type 1 and Type 2 Tri-Hy-NAA-PCs, respectively. (**c**,**d**) Effect of *t*_*An-Top*_, *t*_*An-Bottom*_, and *t*_*pw*_ on FWHM of Type 1 and Type 2 Tri-Hy-NAA-PCs, respectively. (**e**,**f**) Effect of *t*_*An-Top*_, *t*_*An-Bottom*_, and *t*_*pw*_ on interferometric color of Type 1 and Type 2 Tri-Hy-NAA-PCs, respectively.

These conditions are summarized in Table 2 and the obtained result shown in Fig. 9 and Figure S4 (Supporting Information). Figure 9a,b show the dependency of *λ*_*PSB*_ for the different individual PCs composing the structure of Type-1 and Type-2 Tri-Hy-NAA-PCs as a function of *t*_*An-Top*_, *t*_*An-Centre*_, *t*_*An-Bottom*_, and *t*_*pw*_, respectively. It is verified that, likewise in previous cases, *λ*_*PSB*_ undergoes a blue shift with *t*_*pw*_. In the case of Type-1 (Fig. 9a), it is demonstrated that the position of the characteristic PSBs of the APO-NAA-GIFs located at the top and bottom of the Tri-Hy-NAA-PC structures is red-shifted when the relative anodization time (*t*_*An-Top*_ and *t*_*An-Bottom*_) is increased from 5 to 7.5 h. In contrast, the *λ*_*PSB*_ of the central NAA-DBR is red-shifted with decreasing relative anodization time (*t*_*An-Center*_)–from 10 to 5 h, which is in good agreement with the general trend observed in other NAA-based PC structures. Figure 9b shows the obtained results for the Type-2 Tri-Hy-NAA-PCs. From our analysis, we found that the minimum relative anodization time for the central APO-NAA-GIF structure to show its characteristic PSB in the transmission spectrum was *t*_*An-Center*_ = 10 h. The hybrid PC structure produced at *t*_*An-Centre*_ = 5 h did not show characteristic PSB for the APO-NAA-GIF structure. The *λ*_*PSB*_ of the bottom NAA-DBR reveal that a significant red shift occurs when the relative anodization time used to produce this PC (*t*_*An-Bottom*_) is increased from 5 to 7.5 h. In contrast, we observed a slight blue shift in the position of the PSB of the top NAA-DBR structure when *t*_*An-Top*_ is increased from 5 to 7.5 h, which is in good agreement with previous observations.

**Table 2:** Summary table compiling the structural arrangement and fabrication conditions for each Tri-Hy-NAA-PC structure assessed in this study.

Type	Top PC Structure *t*_*An-Top*_ (h)–*T*_*P*_ (s)	Center PC Structure *t*_*An-Center*_ (h)–*T*_*P*_ (s)	Bottom PC Structure *t*_*An-Bottom*_ (h)–*T*_*P*_ (s)	*t*_*An*_ (h)
Type 1	APO-NAA-GIF 6.67 h–700 s	NAA-DBR 6.67 h–1000 s	APO-NAA-GIF 6.67 h–1300 s	20 h
Type 2	NAA-DBR 6.67 h–900 s	APO-NAA-GIF 6.67 h–1100 s	NAA-DBR 6.67 h–1200 s	20 h
Type 1	APO-NAA-GIF 7.5 h–700 s	NAA-DBR 5 h–1000 s	APO-NAA-GIF 7.5 h–1300 s	20 h
Type 1	APO-NAA-GIF 5 h–700 s	NAA-DBR 10 h–1000 s	APO-NAA-GIF 5 h–1300 s	20 h
Type 2	NAA-DBR 7.5 h–900 s	APO-NAA-GIF 5 h–1100 s	NAA-DBR 7.5 h–1200 s	20 h
Type 2	NAA-DBR 5 h–900 s	APO-NAA-GIF 10 h–1100 s	NAA-DBR 5 h–1200 s	20 h

Figure 9c shows the evolution of the FWHM of the PSBs of each individual PC forming the structure of Type-1 Tri-Hy-NAA-PCs as a function of the relative anodization time and *t*_*pw*_. This graph shows that the FWHM of the central NAA-DBR structure is enlarged with decreasing relative anodization time, when *t*_*An-Center*_ is reduced from 10 to 5 h. In the same way than in other NAA-based PCs, a pore widening treatment also enlarges the FWHM. This analysis reveals that, in the case of the top APO-NAA-GIF structure, the FWHM increases as *t*_*An-Top*_ is extended from 5 to 7.5 h. It is also observed that a pore widening treatment increases slightly the FWHM of this PC structure, although in a much less significant manner than in the case of the central NAA-DBR. In contrast, *t*_*An-Bottom*_ did not have any significant effect on the FWHM for the bottom APO-NAA-GIF structure up to *t*_*pw*_ = 2 min. However, at *t*_*pw*_ > 2 min, the FWHM is enlarged with decreasing relative anodization time from 7.5 to 5 h. Likewise in previous Hy-NAA-PCs, the FWHM of the PSB of the central NAA-DBR was found to be broader than that of its APO-NAA-GIFs counterparts. Figure 9d depicts the FWHM of the PSBs of the individual PCs composing the structure of Type-2 Tri-Hy-NAA-PCs. As expected, the FWHM of the NAA-DBRs’ PSB is significantly broader than that of the APO-NAA-GIF, and it is enlarged with *t*_*pw*_. Overall, the FWHM is broader at shorter relative anodization times, for both top and bottom NAA-DBRs. Furthermore, this analysis reveals that the FWHM of the top NAA-DBR is wider than that of its counterpart located at the bottom of the Type-2 Tri-Hy-NAA-PCs.

Figure 9e and f compile digital pictures of the Type-1 and Type-2 Tri-Hy-NAA-PCs assessed in our study. These images denote that the Type-1 Tri-Hy-NAA-PCs (i.e. APO-NAA-GIF top, NAA-DBR center, and APO-NAA-GIF bottom) display clearly defined interferometric colors, which correspond to the position of the PSB of the central NAA-DBR. It is observed that the interferometric color displayed by these PCs undergoes a blue shift when the relative anodization time (*t*_*An-Center*_) is increased from 5 to 10 h. This blue shift is further enhanced with *t*_*pw*_. On the contrary, Type-2 Tri-Hy-NAA-PCs display complex color patterns, which are associated with the position of the PSBs of the two NAA-DBRs embedded in their structure. However, it is also observed that these PCs display well-defined colors when the *λ*_*PSB*_ of one of the NAA-DBRs is in the NIR or UV regions. For instance, the color of the samples produced with *t*_*An-Top*_ = *t*_*An-Bottom*_ = 7.5 h at *t*_*pw*_ = 6 min is green while it turns into orange-yellow when *t*_*An-Top*_ and *t*_*An-Bottom*_ are set to 5 h. It is worthwhile noting that at *t*_*pw*_ = 6 min the PSB of the top NAA-DBR structure is in the UV region of the spectrum and therefore it has no significant contribution to the overall interferometric color of the Tri-Hy-NAA-PC, which is determined by the PSB of the bottom NAA-DBR.

To conclude, this study has presented the first realization of hybrid nanoporous anodic alumina photonic crystals (Hy-NAA-PCs) fabricated by a heterogeneous pulse anodization. This versatile nanofabrication approach enables the precise engineering of the effective medium of NAA in depth to create a set of Hy-NAA-PCs featuring photonic stopbands with precisely engineered features (i.e. number, position, and width) that can be readily tuned across the spectral regions by various anodization parameters. The effects of the anodization period, the relative and total anodization times, the pore widening time, and the arrangement of structures on the optical properties of Hy-NAA-PCs have been established by systematically modifying these parameters and assessing the optical properties of Bi- and Tri-Hy-NAA-PCs. The analysis of the optical properties of Bi- and Tri-Hy-NAA-PCs has revealed that the position of characteristic transmission bands is primarily dependent on the anodization period and the pore widening time, although the relative and total anodization times and the exchange of structures also have impact on these optical features. The assessment of the interferometric colors displayed by the Hy-NAA-PCs has revealed that this optical property is mainly established by the PSBs of the NAA-DBRs composing the structure of Bi- and Tri-Hy-NAA-PCs. Furthermore, we observed that the FWHM of the different individual PCs embedded in the structure of Hy-NAA-PCs follows the characteristics of their mono-structural counterparts, where NAA-DBRs and APO-NAA-GIFs display broad and narrow PSBs, respectively.

In summary, this study has established the first comprehensive rationale for the fabrication of complex photonic crystal structures based on NAA-PCs using a newly developed anodization approach. This versatile nanofabrication method opens new opportunities to develop innovative photonic nanostructures with implications in many fields such as sensing and photonics.

## Methods

### Materials

High purity (99.9997%) aluminum (Al) foils with thicknesses of 0.32 mm were purchased from Goodfellow Cambridge Ltd. (UK) and used to produce Hy-NAA-PCs by HPA process. Sulfuric acid (H_2_SO_4_), phosphoric acid (H_3_PO_4_), hydrochloric acid (HCl), copper (II) chloride (CuCl_2_), and ethanol (EtOH–C_2_H_5_OH) were supplied by Sigma-Aldrich (Australia) and used as received, without additional purification steps. Ultrapure Mili-Q® water (18.2 MΩ cm) was used for the preparation of aqueous solutions in this study.

### Fabrication of Hy-NAA-PCs by HPA

Hy-NAA-PCs were produced by a HPA under galvanostatic mode combining stepwise pulse anodization (STPA)^20^ and apodized sinusoidal pulse anodization (ASPA)^48^. Briefly, before anodization, 1.5 × 1.5 cm Al substrates were cleaned in ethanol and water for 15 min each under sonication, dried under air stream, and electropolished in a mixture of EtOH and HClO_4_ 4:1 (v:v) at 20 V and 5 °C for 3 min to achieve a cleaned mirror-like finishing surface prior to anodization. The electropolished Al substrates were anodized in a 1.1 M aqueous H_2_SO_4_ solution, in which 25 *v*% EtOH was added to prevent the solution from freezing at temperatures below 0 °C^50,51^. HPA was performed in a single step, starting with a constant anodization stage for 1 h at −1 °C at a constant current density of 1.120 mA cm^−2^ to achieve a homogeneous pore growth rate of anodic oxide before switching the anodization mode to HPA. The anodization profile was subsequently set into various arrangements of STPA and ASPA modes, depending on the number and order of structures composing the Hy-NAA-PCs to be produced. The HPA profiles were generated by a custom-designed Labview®-based software following the equations for STPA and ASPA.

For ASPA, the anodization current density was pulsed in a logarithmic negative apodized sinusoidal fashion given by Equation 1:

1 $$J(t)=A{(t)}_{J}.[{\sin }(\frac{2\pi }{{T}_{P}}t)+1]+{J}_{Offset}$$

where *J(t)* is the current density at a given time *t*, *T*_*P*_ is the anodization period, *t*_*An*_ is the total anodization time at ASPA, and *A(t)*_*J*_ is the current density amplitude as a function of time *t* given by Equations 2 and 3:

For t ≤ *t*_*An*_/2,

2 $$A{(t)}_{J}={A}_{{\max }}+(\frac{{A}_{{\min }}-{A}_{{\max }}}{{log}(\frac{{t}_{An}}{2}+10)-1}).({log}(t+10)-1)$$

For t > *t*_*An*_/2,

3 $$A{(t)}_{J}=(\frac{{A}_{{\max }}-{A}_{{\min }}}{{log}({t}_{An}+10)-\,{log}(\frac{{t}_{An}}{2}+10)})\,\times ({log}(t+10)-\,{log}(\frac{{t}_{An}}{2}+10))+{A}_{{\min }}$$

Note that the current density offset (*J*_*offset*_) and the minimum (*A*_*min*_) and maximum amplitude (*A*_*max*_) current density amplitudes were set to 0.280 mA cm^−2^, and 0.000 and 0.210 mA cm^−2^, respectively.

In STPA, the anodization current density was sequentially pulse between high (*J*_*max*_ = 1.120 mA cm^−2^) and low (*J*_*min*_ = *J*_*offset*_ = 0.280 mA cm^−2^) current density values in a stepwise fashion. The number of anodization pulses (*N*_*P*_) set was defined by Equation 4, in which the pulse period (*T*_*P*_) is given by Equation 5:

4 $${N}_{P}=\frac{{t}_{An}}{{T}_{P}}$$

5 $${T}_{P}={t}_{{\max }}+{t}_{{\min }}$$

where *t*_*An*_ is the total anodization time at STPA, and *t*_*max*_ and *t*_*min*_ are the time for maximum and minimum current density pulses, respectively. Note that the time ratio for *t*_*max*_ and *t*_*min*_ (*t*_*max*_:*t*_*min*_) was set to 1:4 for each anodization period.

We assessed the capabilities of HPA to produce Hy-NAA-PCs with well-defined optical properties by modifying the arrangement of individual PC structures and their features through different fabrication parameters such as the total and relative anodization times, the anodization period, and the structural arrangement. Tables 1 and 2 summarize the fabrication conditions used to produce Bi- and Tri-Hy-NAA-PCs in our study. After anodization, the remaining Al substrate in Hy-NAA-PCs was selectively dissolved by wet chemical etching in a saturated solution of HCl–CuCl_2_ using a 5 mm diameter circular window Viton^TM^ mask. The nanoporous structure of Hy-NAA-PCs was subsequently widened by a wet chemical etching treatment in an aqueous H_3_PO_4_ (5 *wt* %) solution at 35 °C under controlled time (*t*_*pw*_), from 0 to 6 min with an interval (*Δt*_*pw*_) of 2 min.

### Optical characterization

The transmission spectra of Hy-NAA-PCs fabricated by HPA under different anodization conditions were acquired at normal incidence (i.e. *θ* = 0°) from 200 to 900 nm with a resolution of 1 nm using a UV-visible-NIR spectrometer (Cary 300, Agilent, USA). Digital images of Hy-NAA-PCs displaying interferometric colors were acquired by a Canon EOS 700D digital camera equipped with a Tamron 90 mm F2.8 VC USD macro mount lens with autofocus feature under natural light illumination.

### Structural characterization

The geometric features of Bi- and Tri-Hy-NAA-PCs were characterized by field emission gun scanning electron microscopy (FEG-SEM FEI Quanta 450). These FEG-SEM images were analyzed by ImageJ (public domain program developed at the RSB of the NIH)^52^.

## Electronic supplementary material

Supplementary Information
