# Supplementary material for: Engineering of Hybrid Nanoporous Anodic Alumina Photonic Crystals by Heterogeneous Pulse Anodization
Source: Sci Rep. 2018 Jun 21;8:9455. doi: 10.1038/s41598-018-27775-6 (PMC6013466; doi:10.1038/s41598-018-27775-6)
Supplement: Supplementary file 1 — Supplementary Information [file 41598_2018_27775_MOESM1_ESM.pdf]

Supporting Information

Engineering of Hybrid Nanoporous Anodic Alumina Photonic Crystals by Heterogeneous Pulse Anodization

Siew Yee Lim [1,2,3], Cheryl Suwen Law [1,2,3], Lluís F. Marsal[4], and Abel Santos [1,2,3,*]

[1]School of Chemical Engineering, The University of Adelaide, Adelaide, SA 5005, Australia
[2]Institute for Photonics and Advanced Sensing (IPAS), The University of Adelaide, 5005 Adelaide, Australia
[3]ARC Centre of Excellence for Nanoscale BioPhotonics (CNBP), The University of Adelaide, 5005 Adelaide, Australia
[4]Department of Electronic, Electric, and Automatics Engineering, Universitat Rovira i Virgili, Avda, Països Catalans 26, Tarragona 43007, Spain

***E-Mail:** abel.santos@adelaide.edu.au

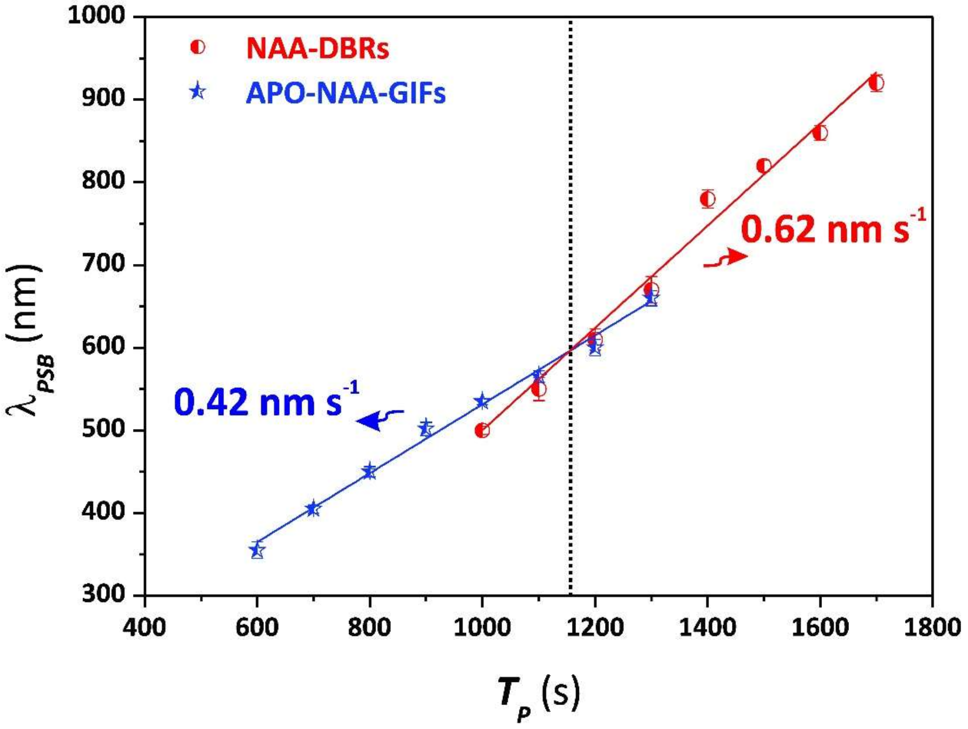

Figure S1. Dependency of λ_{PSB} with T_P for NAA-DBRs and APO-NAA-GIFs and corresponding linear fittings.

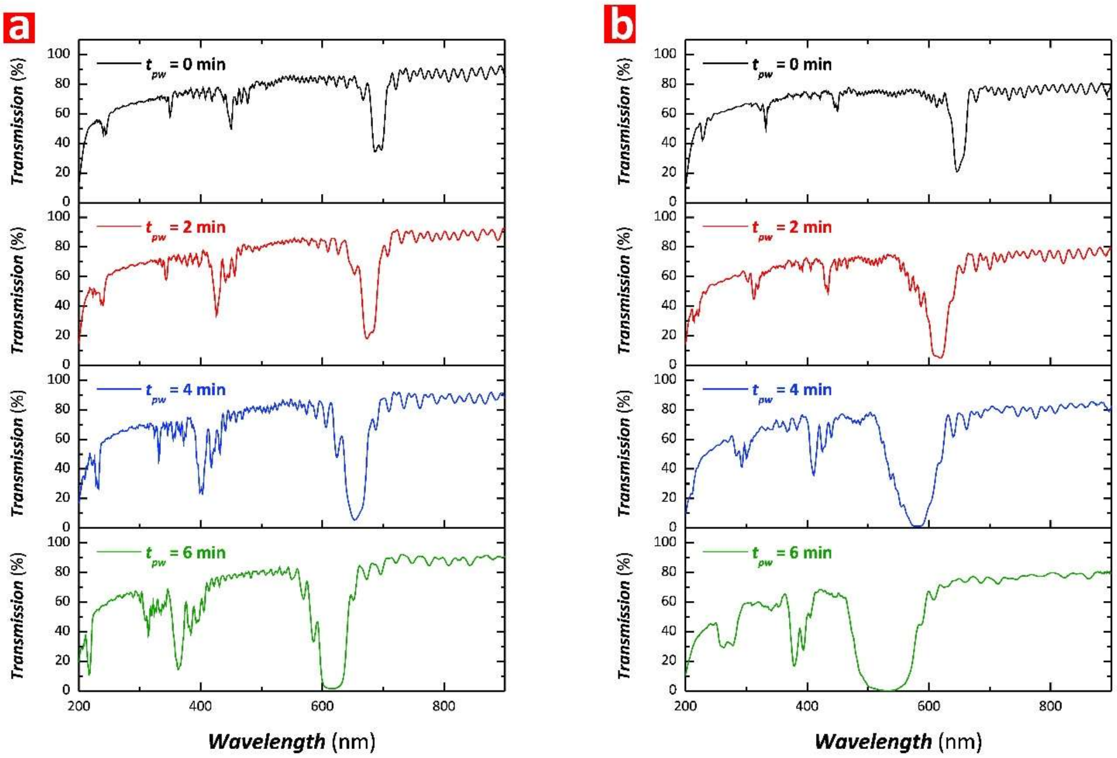

Figure S2. Transmission spectra as a function of t_{pw} for a) Bi-Hy-NAA-PC produced with a top APO-NAA-GIF ($t_{An\text{-}Top}$ = 10 h and T_P = 800 s) and a bottom NAA-DBR ($t_{An\text{-}Bottom}$ = 10 h and T_P = 1200 s), and b) Inv-Bi-Hy-NAA-PC produced with a top NAA-DBR ($t_{An\text{-}Bottom}$ = 10 h and T_P = 1200 s) and a bottom APO-NAA-GIF ($t_{An\text{-}Top}$ = 10 h and T_P = 800 s).

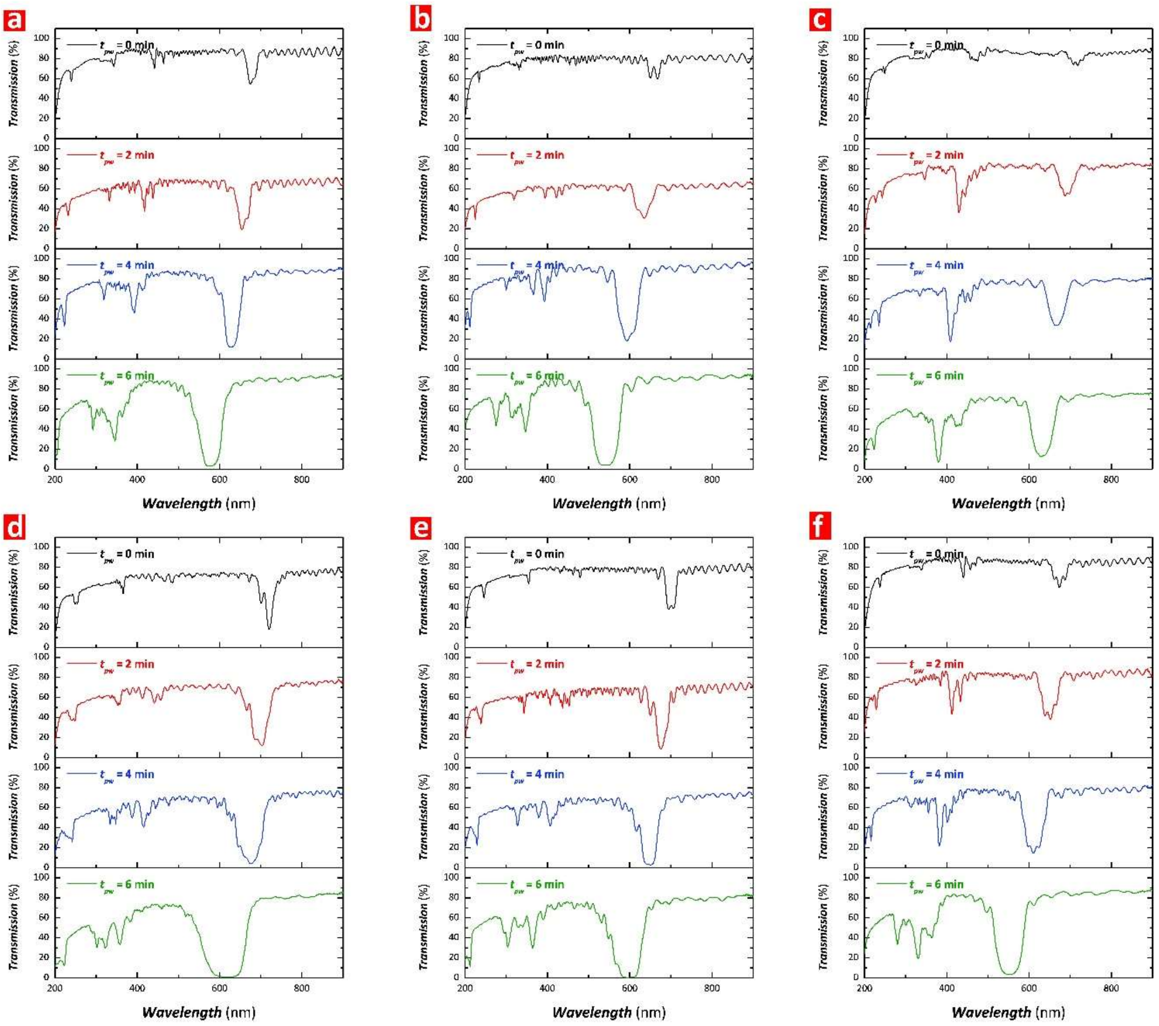

Figure S3. Transmission spectra as a function of t_{pw} for a) Bi-Hy-NAA-PC produced with a top APO-NAA-GIF ($t_{An\text{-}Top}$ = 7.5 h and T_P = 800 s) and a bottom NAA-DBR ($t_{An\text{-}Bottom}$ = 7.5 h and T_P = 1200 s), b) Bi-Hy-NAA-PC produced with a top APO-NAA-GIF ($t_{An\text{-}Top}$ = 5 h and T_P = 800 s) and a bottom NAA-DBR ($t_{An\text{-}Bottom}$ = 5 h and T_P = 1200 s), c) Bi-Hy-NAA-PC produced with a top APO-NAA-GIF ($t_{An\text{-}Top}$ = 15 h and T_P = 800 s) and a bottom NAA-DBR ($t_{An\text{-}Bottom}$ = 5 h and T_P = 1200 s), d) Bi-Hy-NAA-PC produced with a top APO-NAA-GIF ($t_{An\text{-}Top}$ = 5 h and T_P = 800 s) and a bottom NAA-DBR ($t_{An\text{-}Bottom}$ = 15 h and T_P = 1200 s), e) Bi-Hy-NAA-PC produced with a top APO-NAA-GIF ($t_{An\text{-}Top}$ = 5 h and T_P = 800 s) and a bottom NAA-DBR ($t_{An\text{-}Bottom}$ = 10 h and T_P = 1200 s), and f) Bi-Hy-NAA-PC produced with a top APO-NAA-GIF ($t_{An\text{-}Top}$ = 10 h and T_P = 800 s) and a bottom NAA-DBR ($t_{An\text{-}Bottom}$ = 5 h and T_P = 1200 s).

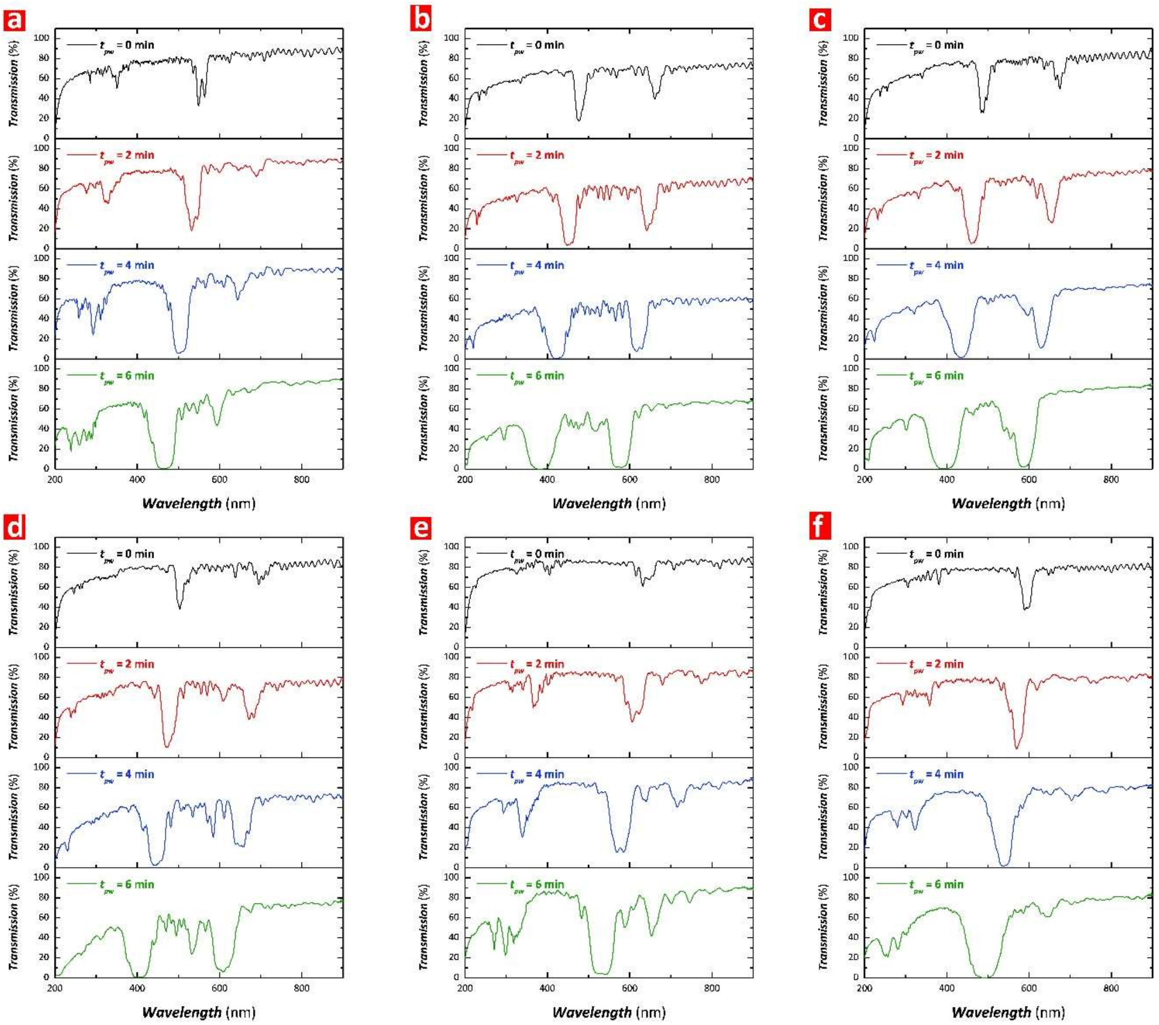

Figure S4. Transmission spectra as a function of t_{pw} for a) Tri-Hy-NAA-PC produced with a top APO-NAA-GIF ($t_{An\text{-}Top}$ = 6.67 h and T_P = 700 s), a central NAA-DBR ($t_{An\text{-}Center}$ = 6.67 h and T_P = 1200 s), and bottom APO-NAA-GIF ($t_{An\text{-}Bottom}$ = 6.67 h and T_P = 1300 s), b) Tri-Hy-NAA-PC produced with a top NAA-DBR ($t_{An\text{-}Top}$ = 6.67 h and T_P = 900 s), a central APO-NAA-GIF ($t_{An\text{-}Center}$ = 6.67 h and T_P = 1100 s), and bottom NAA-DBR ($t_{An\text{-}Bottom}$ = 6.67 h and T_P = 1200 s), c) Tri-Hy-NAA-PC produced with a top NAA-DBR ($t_{An\text{-}Top}$ = 7.5 h and T_P = 900 s), a central APO-NAA-GIF ($t_{An\text{-}Center}$ = 5 h and T_P = 1100 s), and bottom NAA-DBR ($t_{An\text{-}Bottom}$ = 7.5 h and T_P = 1200 s), d) Tri-Hy-NAA-PC produced with a top NAA-DBR ($t_{An\text{-}Top}$ = 5 h and T_P = 900 s), a central APO-NAA-GIF ($t_{An\text{-}Center}$ = 10 h and T_P = 1100 s), and bottom NAA-DBR ($t_{An\text{-}Bottom}$ = 5 h and T_P = 1200 s), e) Tri-Hy-NAA-PC produced with a top APO-NAA-GIF ($t_{An\text{-}Top}$ = 7.5 h and T_P = 700 s), a central NAA-DBR ($t_{An\text{-}Center}$ = 5 h and T_P = 1200 s), and bottom APO-NAA-GIF ($t_{An\text{-}Bottom}$ = 7.5 h and T_P = 1300 s), and Tri-Hy-NAA-PC produced with a top APO-NAA-GIF ($t_{An\text{-}Top}$ = 5 h and T_P = 700 s), a central NAA-DBR ($t_{An\text{-}Center}$ = 10 h and T_P = 1200 s), and bottom APO-NAA-GIF ($t_{An\text{-}Bottom}$ = 5 h and T_P = 1300 s).
